# Beneficial Effects of Kiatsu™ with Ki Training on Episodic Migraine: An Exploratory Study

**DOI:** 10.1155/2021/3290879

**Published:** 2021-10-29

**Authors:** Calvin Y. Tabata, Philip F. Copenhaver, Shirley McCartney, Saman Vazinkhoo, Terry Copperman

**Affiliations:** ^1^Oregon Ki Society, Tigard, OR, USA; ^2^Department of Cell, Developmental and Cancer Biology, Oregon Health & Science University, Portland, OR 97239, USA

## Abstract

**Objective:**

To conduct an exploratory study of Kiatsu^TM^ with Ki training as a potential therapy for treating episodic migraine in women.

**Background:**

Current therapies for migraine have proven partially effective, highlighting the need for alternative treatment options. In preparation for development of a randomized controlled study, the authors conducted a single arm pilot exploratory study to evaluate the effect of Kiatsu with Ki training in adult females with episodic migraine.

**Methods:**

Study subjects established a baseline migraine frequency over 4 weeks. During the following 4 weeks, each subject received instruction in Ki training (to improve concentration, balance, and relaxation), accompanied by Kiatsu (a focused touch method that reduces tension, swelling, and pain). Subjects then participated in one session a month for additional 6 months. The initial session was 1 hour; subsequent sessions averaged 30 minutes. Subjects documented migraine frequency, migraine-specific quality of life scores, and medication use.

**Result:**

Sixty-nine subjects met the study inclusion criteria, and 21 completed the study. Subjects reported a significant reduction in migraine frequency after 1 month (from 7.2 to 3.8 migraines/month; *p* < 0.05), with an overall 53% reduction at 6 months (*p* < 0.001). Significant improvements in quality of life (QoL) were reported after 1 month, with continued improvements until study completion (*p* < 0.0001). A moderate reduction in medication use was also documented (*p* < 0.03), corresponding to improved QoL scores.

**Conclusion:**

Kiatsu with Ki training may be an effective treatment option for females with migraines, either in combination with medications or as a potential alternative to medications for patients who do not benefit from conventional therapies. The results of this pilot study justify the development of a randomized controlled study designed to investigate the potential benefits of this novel therapeutic method for treating migraine.

## 1. Introduction

Migraine is a prevalent neurological disease that affects 37 million people in the US and an estimated 1 billion people worldwide, including more than 9% of men and 20% of women [1]. Migraine is typified by recurrent, often disabling headaches that are often accompanied by neurovascular pathologies that can significantly affect many aspects of work and family life [2]. Conventional prophylactic drugs for treating episodic migraine include beta-blockers, antidepressants, and antiepileptics; however, these medications are only moderately effective (typically reducing migraine frequency by 30−50%) and all have significant side effects that limit their use [3]. Notably, many patients cease using their daily migraine medications within 1 year of starting treatment, due to side effects, personal preference, and cost [4, 5]. Recent therapies targeting calcitonin gene-related peptide (CGRP) or its receptor have provided moderate beneficial effects in a subset of patients (mean reduction in migraine days of 1.5–2 days/month), but their potential long-term side effects remain unknown [6, 7].

A variety of nonpharmacological therapies have also been widely used for migraine treatment, including acupuncture, biofeedback, and cognitive behavioral therapy, also with moderate success [8]. A meta-analysis of 12 studies on acupuncture showed a 25−30% reduction in migraine frequency per month, [9] which was similar to the beneficial effects of riboflavin and oral magnesium [10, 11]. Different forms of biofeedback have provided better responses in specific subsets of patients [12, 13]; however, this modality is usually available only in specialized clinics. In some studies, a combination of pharmacological and behavioral therapies has proven beneficial [14, 15], but migraine sufferers often struggle with adhering to these treatment regimens. Hence, alternative methods are needed to ameliorate migraine in patients who receive only limited benefits from existing treatments [16].

Kiatsu^TM^ therapy is a method developed in Japan by Master Koichi Tohei, the founder of Shin Shin Toitsu Aikido [17]. Kiatsu involves the administration of a focused touch by a trained practitioner, comparable to (but distinct from) acupressure [18, 19]. Kiatsu is applied in conjunction with teaching subjects using the tools provided by “Ki training,” which include methods for postural correction; techniques for deep breathing, relaxation, and meditation; and mind/body exercises designed to improve relaxation and mental focus. During Kiatsu sessions, a skilled practitioner applies moderate pressure to regions of the body that have accumulated stress or inflammation, a method that has been found to relax tight muscles and fascia, reduce swelling and pain, and possibly stimulate natural healing mechanisms. During 25 years, the authors have interacted with numerous adults suffering from migraine, many of whom anecdotally reported a marked decrease in migraine frequency when receiving Kiatsu therapy combined with Ki training. Individuals also reported an increased ability to cope with mental fatigue and stress. These observations are consistent with other studies on the benefits of methods for stress management to reduce migraines [20].

In this report, the authors summarize the results of an exploratory study designed to investigate the potential benefits of Kiatsu therapy for treating episodic migraine in females [21]. Using a single arm pilot protocol, Kiatsu therapy was applied to specific regions of the head, neck, and upper back, as a strategy for reducing accumulated tension in these regions. Concurrently, subjects were trained in methods to improve physical posture, mental focus, and relaxation (components of Ki training), with the goal of mitigating their subsequent accumulation of trigger point and muscle insertion pain. The primary outcome measure was a reduction in the number of migraines per month; subjects were also asked to complete monthly surveys designed to assess quality of life (QoL) and monitor medication use. The positive outcomes of this small pilot study suggest that Kiatsu with Ki training represents a viable treatment option for females suffering from episodic migraine.

## 2. Study Design and Methods

### 2.1. Compliance with Ethical Standards

This study (number 91070114) was approved by the PeaceHealth Institutional Review Board (Lane County, OR). Subjects provided written consent. The study does not meet the National Institutes of Health's definition of clinical intervention (https://grants.nih.gov/policy/clinical-trials/definition.htm) nor does it involve controlled clinical investigations of products subject to US Food and Drug Administration regulation (https://www.fda.gov/science-research/science-and-research-special-topics/clinical-trials-and-human-subject-protection) and it is not registered at clinicaltrials.gov.

### 2.2. Subject Recruitment

Subjects were recruited primarily from family medicine practices in the Eugene-Springfield and Portland areas of Oregon.

### 2.3. Study Design

To minimize the variables in this pilot exploratory study, we focused on females between the age of 18 and 50 years, the most common demographic group afflicted by chronic migraine [22]. Study inclusion criteria were as follows: female subjects aged 18 to 50 years, with a clinical history of migraine headaches (ICD-10; G43.019 migraine without aura and G43.109 migraine 118 with aura corresponding to ICHD-3 migraine codes 1.1 and 1.2) for at least 1 year prior to study onset; a minimum migraine frequency of 4 migraines/month; the ability to provide written informed consent; and commitment to participate in periodic written self-assessments. Exclusion criteria included the following: females younger than 18 years or older than 50 years of age; males; individuals diagnosed with analgesic overuse, headache, or painful cervical nerve compression syndrome; and inability to provide written informed consent. Other exclusion criteria included ongoing use of antipsychotic or antidepressant medications, daily use of benzodiazepines or narcotic medications, drug or alcohol abuse, pregnancy, or plans to become pregnant during the study.

### 2.4. Data Collection

Subjects completed a weekly online diary, in which the number of discrete migraines (defined as severe headache with phonophobia, photophobia, and nausea) was self-reported (Table 1). Migraines that lasted for more than 1 day were tallied as a single event, while migraines that occurred after subjects had been migraine-free for 24 hours were tallied as separate events. Of note is that the duration and intensity of migraine were not quantified in this study. Subjects also completed a validated migraine-specific QoL survey at the onset of the study and at monthly intervals throughout the treatment period (Migraine-Specific Quality of Life Survey questionnaire (MSQOL), 14-question version) [23, 24]. Study subjects recorded the number of migraine medications used each week, independent of dosage. Medications were grouped as follows: (1) over-the-counter analgesics, (2) narcotics, (3) triptans, and (4) preventive migraine medications. Subjects collected 4 weeks of baseline data prior to their first Kiatsu therapy session, thereby serving as their own historical control (Table 1). After this baseline period, subjects underwent four weekly Kiatsu therapy sessions during the first month, followed by additional sessions of Kiatsu once a month for five months; the overall duration of the study was six months. Nonresponders were defined as subjects who exhibited no detectable improvement in all three reporting criteria: (1) migraine frequency, (2) QoL scores, and (3) reduction in medication usage.

### 2.5. Ki Training and Kiatsu Therapy

The initial study visit (session 1) lasted 1 hour, while subsequent sessions were 30 minutes. During session 1, subjects were taught a technique referred to as “Keeping One Point” [25]. This technique involves a precise method for training individuals to sit and stand with good posture while focusing one's mind on their center of balance, with the purpose of improving postural habits and reducing stress. Once learned, this technique can be performed in 3–5 seconds. All subjects readily mastered the technique during their first session, as assessed using physical tests for balance and stability administered by a skilled practitioner (90% of the time provided by CYT, 10% of the time by TC). Once trained, subjects were instructed to practice these techniques at least 100 times per day, as a strategy to reinforce their understanding of the process.

Subjects then received Kiatsu [17] from an advanced practitioner (90% of the time provided by CYT, 10% of the time by TC). During each session, the practitioner applied light/moderate pressure with their hands on different areas of the head, neck, upper back, and shoulder blades. This process was continued until the tight muscles in the neck and back began to relax. Although the duration of each Kiatsu session varied depending on each subject's responsiveness, they typically lasted for an average of ∼15 minutes.

Subjects were also taught a simple meditation exercise called “Ki meditation” [25]. For this method, subjects were instructed to sit comfortably and practice keeping one point (as described above), with the goal of ensuring that they maintained a good posture while focusing on their center of balance. To initiate Ki meditation, subjects were then instructed to follow the imagery of energy moving infinitely in all directions. In addition, subjects were taught a gentle deep breathing technique called “Ki breathing” [26, 27], involving repeated cycles of long, relaxed exhalations (through the mouth) followed by gentle inhalations (through the nose) while being coached to maintain a relaxed posture. After learning these techniques, subjects were instructed to practice Ki meditation for 1−2 minutes followed by Ki breathing for 2−5 minutes before bedtime daily. The goal of this combined exercise was to improve sleep quality [20].

Subjects returned weekly during the first month for a total of four sessions. Instruction in how to correct posture and mental focus (“keeping one point”), practice Ki meditation, and perform Ki breathing was repeated at the beginning of each of these sessions. Kiatsu was also provided during each session, as described above. Subsequently, subjects received additional sessions of Kiatsu once a month for the following 4–5 months (depending on their individual schedules). Subjects documented the number of migraines and medication usage on a weekly basis (Table 1); most subjects also recorded QoL assessments throughout the study.

### 2.6. Statistical Analysis

Sample size determination was based on previous studies examining the effects of acupuncture to treat chronic migraine (migraine frequencies of 12–20/month) [9, 28, 29]. Study primary efficacy endpoint was 6-month migraine and QoL reduction. A single-factor ANOVA (two-tailed *p* value = 0.05; 80% power) determined that a significant reduction in migraine frequency of at least two migraines/month required 16 evaluable subjects at study 6-month endpoint. Data were analyzed using Microsoft Excel. Categorical variables (number of migraines per month, QoL, and medication usage) were assessed for significance using a single-factor ANOVA test with a *p* value of 0.05 for complete data sets. Significance of variations for each month compared to the baseline months was assessed using a two-sample Student's *t*-test assuming equal variances. Data sets with a *p* value of <0.05 for t_stat ≤ t_critical were considered not meeting the null hypothesis (i.e., significant variations were observed). In most cases, *p* values were <0.001.

## 3. Results

### 3.1. Study Population

**Figure 1:**
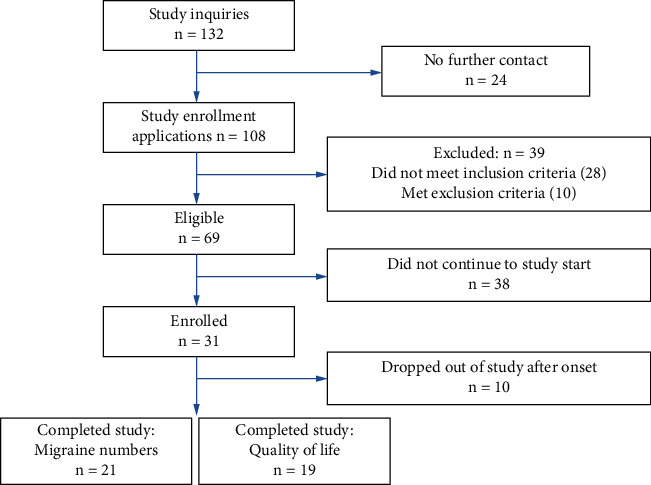
Diagram of participant recruitment and enrollment. Women between 18 and 50 years with a history of episodic migraine were recruited from the local community in the Eugene-Springfield and Portland areas in Oregon. From 132 inquiries, 108 interested individuals proceeded to initial enrollment. 69 potential subjects were deemed eligible (meeting the study criteria and not meeting the exclusion criteria), and 31 subjects subsequently were enrolled. 21 qualified subjects participated in the study, completing their headache logs for at least 6 of the 8 months of the study. Nineteen of these subjects also completed QoL surveys for at least 4 months of the study (see Table 1).

A total of 132 individuals initially inquired about participating in the study, and 108 individuals subsequently applied to enroll (Figure 1). Of this group, 39 individuals did not meet study inclusion criteria or met the exclusion criteria. Of the 69 eligible candidates, 31 subsequently enrolled, while 38 chose not to participate (specific reasons not known). During the study, 10 subjects dropped out prior to completing the treatment sessions. Reasons for drop-out varied; the most common was scheduling and time constraints. All 21 remaining subjects completed the study and kept weekly logs of the number of headaches that they experienced (regardless of duration or intensity). The majority (*n* = 19) also provided monthly QoL assessments for at least 4 months of the study (Table 2). Subjects also recorded medication use throughout the study (frequency; not dosage). Subjects were permitted to modify their medications (based on advice from their medical practitioners), but they were not provided with advice about their individual medication regimen as part of this study. Baseline subject characteristics at study onset (migraine frequency and medication usage) are summarized in Table 1.

Among the 21 subjects who completed the study, one subject showed no detectable improvement in all three reporting criteria (migraine frequency, QoL scores, and reduction in medication usage); of note, this subject had a sustained high migraine frequency of 13 per month and was designated as a nonresponder. Another participant had previously been diagnosed with daily chronic migraines and received Botox injections prior to and during the study, which reduced her diagnosis to episodic migraine. She had 7 migraines/month despite Botox treatment during baseline; MSQOL score was 59. At the study end, she had 5 migraines/month; MSQOL score was 93. A third participant was placed on a CGRP medication during the study by her medical practitioner.

**Figure 2:**
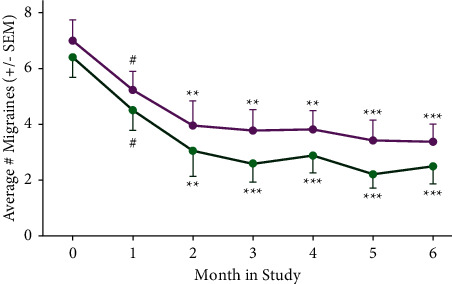
Migraine frequency during the study. Participants recorded the number of migraines per week, independent of intensity or duration. “Month 0” represents baseline data recorded for 4 weeks prior to starting treatment. Magenta curve indicates the average number of migraines experienced by all subjects (*n* = 21). Green curve indicates average number of migraines experienced by all responding subjects (excluding one nonresponder; *n* = 20). ^#^*p*=0.07; ^*∗∗*^*p* < 0.01; ^*∗∗∗*^*p* < 0.001 (Student's unpaired *t*-test).

**Figure 3:**
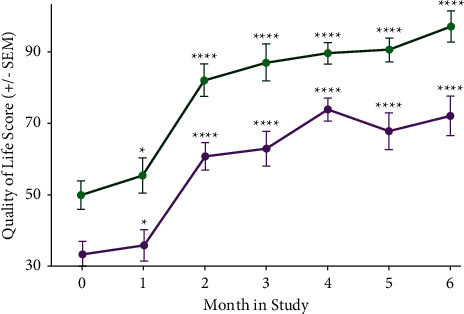
Quality of life (QoL) scores reported by subjects (calculated using the MSQOL survey tool) during the study. The magenta curve indicates the average QoL scores experienced by all subjects who reported scores for at least 4 months of the study (*n* = 19). The green curve indicates the average QoL scores experienced by subjects (as defined in methods; *n* = 16). ^*∗*^*p* < 0.05; ^*∗∗∗∗*^*p* < 0.0001 (Student's unpaired *t*-test).

**Figure 4:**
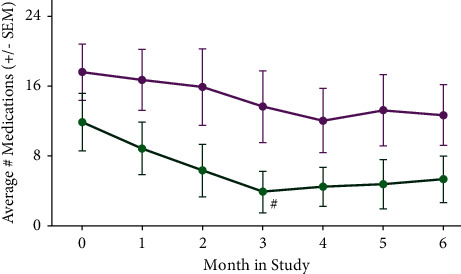
Average medication taken by subjects for migraine during the study. Participants reported the number of medications used per week, regardless of dosage. The magenta curve indicates average medication use by all responding subjects, including the nonresponders (*n* = 21). The green curve indicates the average medication use by subjects (as defined in methods; *n* = 18). ^#^*p*=0.06 (Student's unpaired *t*-test).

We compared the combined responses of the 21 subjects who completed the study with combined subject responses that omitted the three subjects, which were relevant and noted above, as shown in Figures 1–4.

### 3.2. Primary Outcome Measure: Effects of Kiatsu and Ki Training on Migraine Frequency

Based on daily migraine diaries, most subjects experienced a significant (*p* < 0.01) reduction in the number of migraines after 2 months of therapy, and all subjects (except for the nonresponders) continued to experience fewer migraines each month by the end of the study (*p* < 0.001; unpaired *t*-tests; Figure 2). “Month 0” represents baseline data recorded for 4 weeks prior to starting treatment. On average, all subjects experienced a 53% reduction in the number of migraines compared to their initial levels, while 7 subjects experienced a 100% reduction. The percentage of participants who had at least a 50% reduction of in migraine frequency was 65%.

### 3.3. Secondary Outcome Measure: Effects of Kiatsu and Ki Training on Quality of Life

Subjects also reported a marked improvement in their overall QoL, as measured using the validated MSQOL survey instrument (Figure 3). Most subjects reported a detectable improvement as early as study month 1 (*p* < 0.05), with an average increase of 30 QoL points by month 2. Moreover, average QoL values continued to improve significantly throughout the duration of the study (*p* < 0.0001; Figure 3). Of note, all subjects consistently reported improvements in their ability to carry out daily tasks, engage positively in family activities, and perform better at work (personal communication).

### 3.4. Additional Outcome Measure: Effects of Kiatsu and Ki Training on Medication Usage

Although a reduction in medication use was not a primary objective of this study (subjects were encouraged to follow instructions from their individual providers), subjects did record the number of times they used migraine-related medications, independent of dosage. A moderate but steady decrease in the average number of medications taken over the course of the study that approached significance by month 3 was noted (Figure 4, green curve; *p*=0.06), with some minor fluctuations in the subsequent months. Given our evidence that a combination of Kiatsu therapy with Ki training exercises reduced migraine frequency, it would be expected that subjects would voluntarily reduce their medication use. Whether this beneficial response might involve selective reductions in certain classes of medications (or a transition to alternative medications with fewer side effects) was not examined in this study, but will be a focus of future investigations.

### 3.5. Adverse Events

No adverse events were reported or observed.

## 4. Discussion

Migraines are triggered by a variety of factors, including stress, certain foods, disturbed sleep, and changes in menstrual cycles [30]. Drugs with established efficacy for preventing migraine include amitriptyline, topiramate, valproic acid, metoprolol, and propranolol [31, 32]. However, treatment with these medications typically results in reductions in migraine frequency of only 35−50%, with adverse side effects reported by 15−30% of all patients that often cause them to discontinue treatment. Hence, there remains an unmet need for alternative strategies that can alleviate migraine without debilitating side effects.

This single arm pilot study explored a novel strategy for preventing migraine, which involved the integration of Kiatsu therapy with Ki training methods designed to improve and reduce stress. For this approach, we incorporated instruction in how to achieve and maintain good posture, practice Ki meditation, and use daily Ki breathing methods, combined with periodic sessions of Kiatsu therapy. As summarized above, the results of this study were encouraging; of the 21 subjects who completed the study, 19 participants experienced a 53% average reduction in migraine frequency. Moreover, subjects reported no adverse side effects, in contrast to the well-documented side effects of most migraine medications [16, 33]. Notably, the reduction in migraine frequency observed in the study (an average reduction of 4 migraines per month) compares favorably with a previous study testing the effects of other relaxation and exercise methods, which produced mean reductions of 0.97 and 0.83 migraines during the final month of the protocol (comparable to the effects of topiramate) [14].

The largest positive impact of Kiatsu therapy with Ki training was on QoL (as recorded using the MSQOL survey tool), with a significant effect detectable as early as study month 1 (*p* < 0.05) and continued improvement through the duration of the study (*p* < 0.0001), even after the frequency of Kiatsu sessions had decreased to one per month. Subjects also consistently reported an increased ability to carry out daily tasks, perform better at work, and be positively involved in family activities (personal communication). In the current study, it was not possible to distinguish between the beneficial effects of Kiatsu therapy alone from the overall benefit of Ki training methods (including meditation and Ki breathing). Although all subjects noted improvements in their condition immediately following each Kiatsu therapy session, we attribute the sustained improvement in QoL scores to their continued use of the Ki training methods for maintaining good posture and reducing stress. These positive outcomes suggest that for females who may not benefit from conventional pharmacological treatments (or prefer to avoid medication use), Kiatsu with Ki training may represent an effective alternative treatment option.

In our experience, individuals who have been experiencing multiple severe migraines per month find it difficult to significantly improve their condition with Ki training alone. The reverse has also been observed, whereby Kiatsu therapy initially reduced migraine frequency, but without sustained Ki training and practice, the individual's condition gradually worsened (unpublished observations). In contrast, the results of the current study show that Kiatsu in combination with Ki training methods was effective in improving the status of most subjects to the point that they could sustain the benefits of this therapy with their ongoing daily practice.

It would be remiss not to note that the methods used in the study protocol required a significant time commitment by both the practitioners and subjects alike. Other types of physical and chiropractic therapy requiring similar time commitments have also been shown to provide some benefit to migraine sufferers, although the results have been more modest [34, 35]. Likewise, a variety of methods combining pharmacotherapy with biofeedback, relaxation training, and cognitive behavior therapy have provided moderate improvements for some groups, but the cost and commitment required for these strategies can be prohibitive for many patients [36]. Although there has been renewed interest in the use of acupuncture to treat episodic migraine [29], a recent meta-analysis of 12 studies on acupuncture showed only a 25−30% reduction in migraine frequency per month, [9] similar to the benefits of riboflavin or oral magnesium alone [10, 11]. Equally important, lasting improvement beyond the initial treatment period was not achieved in many of these trials [28].

In the current study, no attempt was made to optimize medical management. Nevertheless, subjects reported a reduction in their medication use over six months of their participation (Figure 4), consistent with the increase in QoL scores (Figure 3). In future studies, it will be important to evaluate how Kiatsu with Ki training affects both the dosage and frequency of medication use and whether subjects are able to transition to medications with fewer adverse side effects in response to this therapeutic strategy.

### 4.1. Remaining Questions

Our study raises important questions regarding how Kiatsu with Ki training functionally improves migraine behavior. What is the effect of physical posture and postural self-correction on the migraine diathesis? Recent evidence suggests that migraine might be associated with disruptions affecting the drainage of cerebral spinal fluid via the brain's glymphatic system [37, 38]. By reducing tightness in the upper back and neck, Kiatsu therapy might positively affect this process. Likewise, migraine has been postulated to involve neurogenic neuroinflammation associated with trigeminovascular activation [39, 40]. Based on the results of this study, we postulate that Kiatsu might also mitigate this type of inflammatory response, albeit via mechanisms that remain to be explored.

### 4.2. Study Limitations and Future Directions

The placebo effect, which can help patients to maximize the healing effect of an intervention through positive attitudes and beliefs, is essential to many medical interactions [41, 42]. As such, the placebo effect associated with our study cannot be overlooked. Placebo effects not accompanied by any substantial physiologic effects would be expected to diminish significantly over the 6 months of the study. The improvements in the frequency of migraine and MSQOL were sustained throughout the study, making it unlikely that the results were unduly related to a placebo effect. Using Kiatsu with Ki training to treat migraines involves practices designed to employ the power of the mind to positively influence the body. For example, subjects were instructed to correct their posture (by “keeping one point”) many times a day, in addition to daily practice of mediation and breathing relaxation methods. The combination of frequent self-adjustment of posture and mental relaxation is integral to the methodology used for this study and has clearly contributed to the beneficial effects of Kiatsu.

Individual subject's psychological profile and previous prophylactic treatment data were not collected as part of the study and are a study limitation.

For the current analysis, subjects recorded only severe migraines (with phonophobia and photophobia), whereas tension-type headaches and migraines with visual aura were not included. Although the number of discrete migraines was tracked throughout the study, the number of migraine days (and migraine intensities) was not recorded. Likewise, the frequency of migraine medication use was routinely recorded, but more detailed information about alterations in medication usage or other potential comorbidities that might affect outcomes was not collected. Lastly, due to the exploratory pilot nature of this study, no concomitant control group was included, nor was the study designed to directly compare the protocol with other preventive therapies. In future research, we intend to investigate whether combining active medication management with Kiatsu therapy might produce an even greater benefit to migraine sufferers.

The results of this pilot exploratory study will also provide the framework for developing a randomized controlled clinical study to test further the potential benefits of Kiatsu to Ki training for treating different forms of migraine.

## 5. Conclusion

The results of this single arm pilot study indicate that the combination of Ki training methods with Kiatsu represents a promising new approach for providing lasting benefits for females suffering from migraine. Specifically, application of Kiatsu therapy (by a trained practitioner) combined with Ki training exercises (performed daily by subjects) significantly reduced migraine frequency while improving QoL scores and was associated with a moderate reduction in medication use. We propose that this combined approach may therefore be beneficial for treating other patient groups (including both men and women of different ages) and for patients suffering from additional types of headaches, including recurrent tension-type headache, cervicogenic headache, and migraine with visual aura.

## Figures and Tables

**Table 1:** Subject baseline characteristics (weekly migraine number and medications over 4 weeks; *n* = 21).

	Baseline data (collected over 4 weeks)				
Subject ID	Weekly migraine (*n*)	Medications (*n*)			
		Over-the-counter	Narcotics	Triptans	Preventive
2	6	14	0	3	28
4	3	0	0	2	0
6	13	16	0	6	14
7	4	1	0	0	1
8	7	7	4	8	0
9^botox^	6	2	0	5	0
13	4	2	0	0	0
15	5	17	4	0	0
16	6	14	0	7	0
17	11	24	0	11	0
19	4	0	8	0	0
20	10	17	1	0	0
21	5	0	0	0	0
22	10	5	0	7	0
23	9	14	0	10	1
28	6	0	0	12	0
30	5	2	0	2	1
31	15	18	0	13	0
32	12	48	0	0	7
1	11	5	12	0	0
18	3	23	8	0	0

^botox^One subject reported Botox use.

**Table 2:** Summary of participants who reported migraine frequency for at least 6 months of the study (left column) and quality of life scores for at least 4 months of the study (right column).

Number of migraines		Quality of life	
Subject #	Data available; months (*n*)	Subject #	Data available; months (*n*)
2	7	6	7
4	7	9	7
6	7	17	7
7	7	4	6
8	7	7	6
9	7	8	6
13	7	13	6
15	7	15	6
16	7	16	6
17	7	19	6
19	7	20	6
20	7	28	6
21	7	31	6
22	7	2	5
23	7	22	5
28	7	30	5
30	7	21	4
31	7	26	4
32	7	32	4
1	6
18	6		
Total (n)	21	19	

Number of migraines ≥6 months of data (*n* = 21). Quality of life ≥4 months (*n* = 19).

## Data Availability

Data can be made available upon request.

## Abbreviations

CGRP: Calcitonin gene-related peptide
MSQOL: Migraine-specific quality of life survey
QoL: Quality of life.
